# Plasma ceramides as biomarkers for microvascular disease and clinical outcomes in diabetes and myocardial infarction

**DOI:** 10.1186/s40842-024-00186-5

**Published:** 2024-09-17

**Authors:** Debora Leonor Junqueira, Alexandre Biasi Cavalcanti, Juliana Maria Ferraz Sallum, Erika Yasaki, Isabella de Andrade Jesuíno, Alline Stach, Karina Negrelli, Leila de Oliveira Silva, Marcela Almeida Lopes, Adriano Caixeta, Mark YY Chan, Jianhong Ching, Valdemir Malechco Carvalho, Andrea Tedesco Faccio, Jeane Tsutsui, Edgar Rizzatti, Rafael Almeida Fonseca, Scott Summers, Henrique Almeida Fonseca, Carlos Eduardo Rochitte, José Eduardo Krieger, Leonardo Pinto de Carvalho

**Affiliations:** 1 Heart Hospital-HCOR, Desembargador Eliseu Guilherme, N° 147, Paraíso, São Paulo, CEP: 04004-030 Brazil; 2 Federal University of São Paulo State-UNIFESP, Rua Napoleão de Barros, N° 715, Vila Clementino, São Paulo, CEP: 04004-030, Brazil; 3 Yong Loo-Lin School of Medicine, Cardiac Department, National University of Singapore, NUHCS, 1E Kent Ridge Road, NUHS Tower Block, Level 9, Singapore, 119228 Singapore; 4 Duke-NUS Graduate Medical School, Metabolomics Research Center, 8 College Rd, Singapore, 169857 Singapore; 5 Fleury Group, Av. Santo Amaro, N° 4584, Brooklin, São Paulo, 04702-000 Brazil; 6 Heart Institute-InCor, University of São Paulo Medical School Hospital, Av. Dr. Eneas de Carvalho Aguiar, N° 44, Cerqueira Cesar, São Paulo, CEP: 05403-900 Brazil; 7 Department of Nutrition and Integrative Physiology and the Diabetes and Metabolism Center, University of Utah, 250 1850 E, Salt Lake City, UT 84112 USA

**Keywords:** Diabetic, Microvascular disease, Ceramides, And myocardial infarction

## Abstract

**Background:**

Ceramides have recently been identified as novel biomarkers associated with diabetes mellitus (DM) and major adverse cardiac and cerebrovascular events (MACCE). This study aims to explore their utility in diagnosing microvascular disease.

**Methods:**

This study prospectively enrolled 309 patients from 2018 to 2020 into three groups: healthy controls (Group 1, *N* = 51), DM patients without acute myocardial infarction (AMI) (Group 2, *N* = 150), and DM patients with AMI (Group 3, *N* = 108). We assessed outcomes using stress perfusion cardiac magnetic resonance (CMR) imaging for coronary microvascular disease (CMD) (Outcome 1), retinography for retinal microvascular disease (RMD) (Outcome 2), both CMD and RMD (Outcome 3), and absence of microvascular disease (w/o MD) (outcome 4). We evaluated the classification performance of ceramides using receiver operating characteristic (ROC) analysis and multiple logistic regression. 11-ceramide panel previously identified by our research group as related to macrovascular disease were used.

**Results:**

Average glycated hemoglobin (HbA1c) values were 5.1% in Group 1, 8.3% in Group 2, and 7.6% in Group 3. Within the cohort, CMD was present in 59.5% of patients, RMD in 25.8%, both CMD and RMD in 18.8%, and w/o MD in 38.5%. The AUC values for the reference ceramide ratios were as follows: CMD at 0.66 (*p* = 0.012), RMD at 0.61 (*p* = 0.248), CMD & RMD at 0.64 (*p* = 0.282), and w/o MD at 0.67 (*p* = 0.010). In contrast, the AUC values using 11-ceramide panel showed significant improvement in the outcomes prediction: CMD at 0.81 (*p* = 0.001), RMD at 0.73 (*p* = 0.010), CMD & RMD at 0.73 (*p* = 0.04), and w/o MD at 0.83 (*p* = 0.010). Additionally, the plasma concentration of C14.0 was notably higher in the w/o MD group (*p* < 0.001).

**Conclusions:**

Plasma ceramides serve as potential predictors for health status and microvascular disease phenotypes in diabetic patients.

**Supplementary Information:**

The online version contains supplementary material available at 10.1186/s40842-024-00186-5.

## Background

Diabetes mellitus (DM) is a growing global health concern, with projections indicating a steady increase in its prevalence worldwide. In 2019, an estimated 463 million individuals were reportedly living with diabetes, accounting for 9.3% of the global adult population [1]. These numbers are expected to rise to 578 million (10.2%) by 2030 and 700 million (10.9%) by 2045 [1], respectively.

The complications arising from hyperglycemia can be broadly categorized into macrovascular and microvascular diseases. The latter, affecting small blood vessels, includes long-term complications that can have a detrimental impact on vascular and endothelial functions. Notably, studies have suggested that microvascular and macrovascular complications share underlying pathophysiological similarities [2], such as impaired endothelial function, inflammation, neovascularization, apoptosis, and a hypercoagulable state [3, 4]. On the other hand, a healthy endothelium facilitates vasodilation, exhibits atheroprotective properties through antioxidant and anti-inflammatory effects, possesses anticoagulant and profibrinolytic effects, and mitigates vascular permeability [5].

Retinal microvascular disease (RMD) is a diabetes-associated complication, generally categorized as either proliferative or non-proliferative. RMD is present in 30% of patients with angina and correlates with elevated morbidity and mortality rates [6]. Conversely, coronary microvascular disease (CMD) is often underdiagnosed; however, new diagnostic methods with higher accuracy and efficiency have been developed in recent years [7].

Mass spectrometry (MS) has proven to be a promising tool for biomarker identification. Prior studies underscore the association between ceramides and major cardiovascular and cerebrovascular adverse events (MACCE) [8]. Ceramides, which are among the most bioactive membrane lipids, regulate signal transduction pathways pivotal for cell survival or death [9]. Ceramides are lipids formed from fatty acids, playing a fundamental role in the cell membrane and influencing enzymatic activity. Their accumulation in tissues such as skeletal muscles and adipose tissue is associated with insulin resistance and the development of diabetes [10].

Notably, ceramides accumulate within coronary atheromatous plaques [11], and their glycosylated variants, such as glucosylceramides and lactosylceramides, are more abundant in regions of arterial tissue with discernible plaque development [2]. In recent years, there has been a growing interest in understanding the molecular mechanisms that regulate ceramide biosynthesis in the heart [12]. Elevated concentrations of ceramide lipids in blood plasma and tissues correlate strongly with the onset of type 2 diabetes (T2D), hepatic steatosis, and cardiovascular diseases [13, 14]. These correlations arise from processes such as lipotoxicity, insulin resistance, and complications in both macrovascular and microvascular systems. Several studies indicate that ceramides impair mitochondrial function in a wide range of experimental systems, including the kidney [15].

Our study hypothesis is that ceramides measured by MS can be used as a noninvasive method for diabetic microvascular disease diagnosis.

## Methods

### Study design

This prospective, multicenter, national observational study was conducted in Brazil, enrolling 258 diabetic patients and 51 healthy individuals. Two centers were selected based on the profiles of the required patients and the availability of equipment for conducting the exams. In compliance with the Helsinki Declaration [16], the study obtained approval for data collection and analysis from the domain-specific institutional review boards of all participating hospitals. The primary aim was to enhance the clinical care of diabetic patients through early and noninvasive identification of plasma ceramides correlated with microvascular dysfunction. Prior to participation, all individuals provided signed informed consent. The HCOR Research Institute oversaw centralized data collection and site monitoring to ensure protocol compliance.

### Study population

From 2018 to 2022, 258 diabetic patients eligible for retinography, stress cardiovascular magnetic resonance (CMR), and blood collection were prospectively recruited. An additional 51 participants comprised the healthy control group (Group 1), undergoing echocardiogram assessments to evaluate heart function instead of stress CMR.

**Fig. 1:**
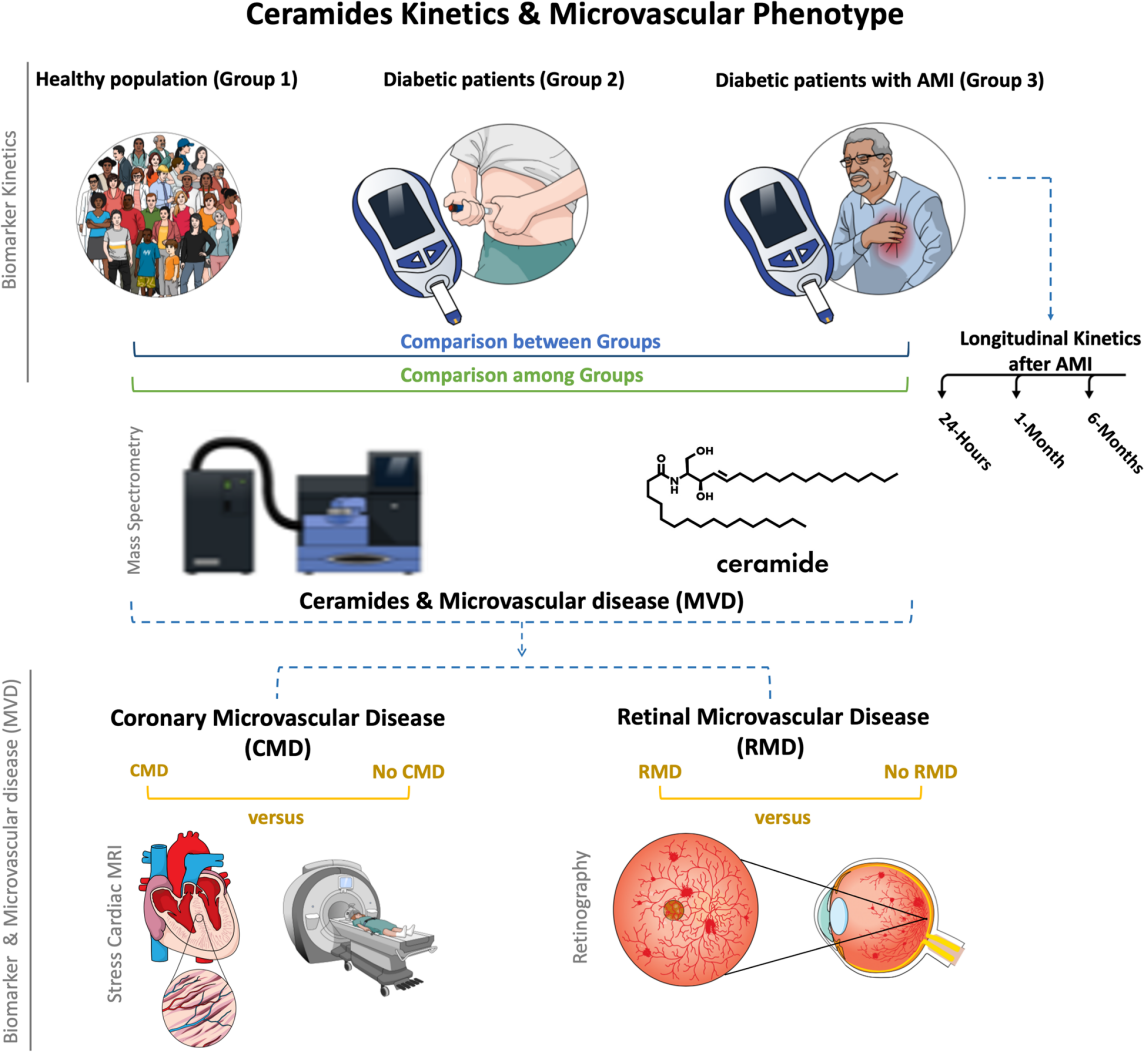
Study workflow

Diabetic patients were stratified based on their acute myocardial infarction (AMI) status: 150 patients without AMI comprised Group 2, while 108 patients with AMI formed Group 3. All Group 3 patients underwent coronary angiography as part of their acute coronary syndrome (ACS) treatment regimen. All patients were fully revascularized with obstructive lesions excluded via angiography and/or Fractional Flow Reserve assessments. Additionally, microvascular resistance was assessed using coronary flow reserve (CFR). Within Group 3, 50 patients underwent triplicate blood collection at intervals of 24 h, 1 month, and 6 months post-AMI for longitudinal kinetic analysis, resulting in the exclusion of two patients lacking triplicate blood samples. Figure 1 outlines the study workflow. Recruitment strategies varied: Groups 1 and 2 participants were sourced from online communities or personal networks, whereas Group 3 individuals were enrolled during the hospitalization phase for acute AMI care.

The inclusion criteria for this study required participants to be over 21 years old, diagnosed with type 1 or 2 diabetes according to the American Diabetes Association (ADA) criteria [17] (for Groups 2 and 3), and have experienced an AMI as per the Fourth AMI Universal definition [18] (Group 3). Group 1 members were required to have normal-range laboratory results from a comprehensive set of tests, including an echocardiogram and retinography. Importantly, they remained free of illnesses throughout the six-month follow-up, thereby preventing the need for CMR due to its futility.

Exclusion criteria included active cancer, glaucoma, prior stroke, severe renal impairment (eGFR < 30 ml/m), uncontrolled hypertension (systolic blood pressure ≥ 160 mmHg and/or diastolic blood pressure ≥ 90 mmHg), uncontrolled dyslipidemia (LDL > 150 mg/dL despite ongoing treatment), or an inability to undergo retinography or stress CMR.

### Clinical and laboratory data

Clinical and laboratory data were categorized into various domains, including clinical history, socioeconomic factors, physical examinations, comorbidities, current medications, and treatment adherence. The Morisky Medication Adherence Scale (MMAS) Questionnaire [19] was employed as a tool to evaluate patient adherence to prescribed drug treatment. The test comprises a four-question questionnaire designed to gauge patients’ consistency in adhering to prescribed medication, with responses gathered during face-to-face medical interviews.

The cohort was analyzed based on four outcomes to assess patients across different spectrums of disease progression: non-diabetic and diabetic patients with and without microvascular and macrovascular disease. However, only patients able to perform CMR, retinography, or both were incorporated into the outcome analysis. Some patients were precluded from undergoing these tests due to conditions such as claustrophobia or pandemic-related restrictions.

### Study procedures

Stress-induced myocardial ischemia (SIMI) was assessed using cardiovascular magnetic resonance imaging (CMR) on a 1.5T scanner (GE 450). The CMR examination was conducted on 114 patients from Group 2 and 45 patients from Group 3 following a standard protocol. This protocol included the acquisition of LV short and long-axis cine images (using a steady-state free precession – SSFP sequence) and late gadolinium enhancement.

First-pass myocardial perfusion was captured in the LV short-axis plane, obtained two to three minutes after pharmacological stress using dipyridamole at 0.56mg.kg^−1^ injected over four minutes. A single dose of 0.05 mM.kg^−1^ of a nonionic, low-osmolar Gd-based contrast agent was then injected into the antecubital vein using a power injector at a rate of 5mL.s^−1^, followed by a 20mL saline flush. Immediately after the stress perfusion image sequence, aminophylline was administered intravenously. The heart was divided into 17 segments for myocardium perfusion assessment [20], which was determined not only in the injected ischemic segment but also in non-injected myocardial segments; each segment was scored as presenting normal perfusion (0), mild [1], moderate [2], or severe [3] perfusion defect. Coronary flow reserve velocity is a reliable measure of coronary microvascular function in the absence of any epicardial flow limitation, and cutoff values ≤ 2.5 are commonly used as indicative of impaired coronary microvascular function [20].

### Acquisition of phase contrast cine CMR data from the coronary sinus

All CMR exams were analyzed using a dedicated CMR-TT software (CVi42 5.13.5, Circle Cardiovascular Imaging Calgary, Canada), which allows flow 2D parameter analysis based on phase contrast cine images to quantify blood flow in the coronary sinus.

The imaging plane for the acquisition of coronary sinus flow was adjusted to be perpendicular to the coronary sinus at a specific distance from its ostium on axial cine CMR images [21].

The contours of the coronary sinus were manually traced, phase by phase, throughout the entire cardiac cycle. Subsequently, a phase-offset correction for the method of static tissue mask in background correction was performed using the CVi42 software. Blood flow in the coronary sinus was calculated by integrating the product of the cross-sectional area and mean velocity in the coronary sinus, then corrected using mean velocity in the adjacent tissue for all cardiac phases in the cardiac cycle. Myocardial blood flow (MBF) was calculated as follows:

$$\mathrm{MBF}\;(\mathrm{ml}/\min/\mathrm g)\:=\:\mathrm{coronary}\;\mathrm{sinus}\;\mathrm{flow}\;(\mathrm{ml}/\min)/\;\mathrm{LV}\;\mathrm{mass}\;(\mathrm g)$$

Resting MBF correlates linearly with the rate pressure product at rest, but hyperemic MBF does not correlate linearly with the rate pressure product [22]. Therefore, we corrected the resting MBF by resting rate pressure product at rest from each subject using the following formulas (MBF during infusion dipyridamole was not corrected by rate pressure product during stress) [21].

$$\mathrm{Corrected}\;\mathrm{MBF}\;\mathrm{at}\;\mathrm{rest}\;(\mathrm{ml}/\min/\mathrm g/)\:=\:\mathrm{MBF}\;\mathrm{at}\;\mathrm{rest}\;(\mathrm{ml}/\min/\mathrm g)\;/\;\mathrm{rate}\;\mathrm{pressure}\;\mathrm{product}\;\mathrm{at}\;\mathrm{rest}\;(\mathrm{mm}\;\mathrm{Hg}/\mathrm{mim})\;\mathrm x\;7,500$$

$$\mathrm{Rate}\;\mathrm{pressure}\;\mathrm{at}\;\mathrm{rest}\;(\mathrm{mm}\;\mathrm{Hg}/\min)\:=\:\mathrm{systolic}\;\mathrm{blood}\;\mathrm{pressure}\;\mathrm{at}\;\mathrm{rest}\;(\mathrm{mm}\;\mathrm{Hg})\;\mathrm x\;\mathrm{heart}\;\mathrm{rate}\;\mathrm{at}\;\mathrm{rest}\;(\mathrm{beats}/\min)$$

The average rate pressure product at rest was 7,500 from healthy controls with a mean age of 50.1 ± 9.7 years, as reported in a previous study [22, 23].

Δ MBF and coronary flow reserve (CFR) were calculated as:

$$\mathrm\Delta\;\mathrm{MBF}\;(\mathrm{ml}/\min/\mathrm g)\:=\:\mathrm{MBF}\;\mathrm{during}\;\mathrm{infusion}\;\mathrm{dipyridamole}\;(\mathrm{ml}/\min/\mathrm g)\;-\;\mathrm{corrected}\;\mathrm{MBF}\;\mathrm{at}\;\mathrm{rest}\;(\mathrm{ml}/\min/\mathrm g)$$

### Sample preparation and Mass Spectroscopy (MS) analysis

Analytes were extracted from plasma by adding a solution composed of ethanol containing 0.1% ammonium hydroxide (v/v) and deuterated internal standards followed by sonication and agitation. For MS analysis, the Transcend TLX-4 system was utilized, which consisted of four Dionex UltiMate 3000 quaternary pumps, four Dionex UltiMate 3000 binary pumps, one VIM, and one CTC PAL autosampler. The system was coupled to a TSQ Altis Triple Quadrupole Mass Spectrometer with a heated-electrospray ionization (HESI) source from Thermo Fisher Scientific, San Jose, CA, USA. Two TurboFlow Cyclone-C8 XL 0.5 × 50 mm columns and two Accucore C30 50 × 2.1 mm columns from Thermo Fisher Scientific were used in the TLX-4 system.

For the first dimension (TurboFlow chromatography), the mobile phase A consisted of water with 0.1% formic acid, mobile phase B was ethanol, and mobile phase C was acetonitrile/isopropanol/acetone (40:40:20, v/v). For the second dimension, mobile phase A was H_2_O/acetonitrile/methanol/formic acid (56:14:30:0.1, v/v), and mobile phase B was isopropanol/acetonitrile/formic acid (90:10:0.1, v/v). The extracts were injected into the system, and detection was achieved by monitoring protonated precursor ions and their respective fragments (264.27 for ceramides, 266.28 for dihydroceramide, 271.31 for ceramides-d7, and [M + H-H_2_O] + for qualifier transitions of ceramides and dihydroceramide).

### Statistical analysis

The data were expressed as mean ± SD for continuous variables that were normally distributed or median (range) for skewed data. Percentages were presented for categorical variables. Comparisons among group variables were performed using the Fisher Exact Test for categorical variables, ANOVA for parametric continuous variables, or the Kruskal-Wallis Test for non-parametric continuous variables. Three ceramide ratios associated with pre-diabetes in a large populational study [24] were selected for benchmark analysis, and mean ± SD were compared between patients with and without diabetes in our cohort to confirm this association: ceramide ratios C18.0/C16.0; C18.0/C24.0, and C18.0/C24.1. All study outcomes were compared using the ROC Curve between the three ceramide ratios and the 11-ceramide panel discovered by our group previously [14]. The individual ceramides used to develop the three ceramide ratios were also included in the panel of 11 ceramides.

All outcomes were analyzed with each of the 11 ceramides by multiple logistic regression, and significant ceramides were selected to build the study conclusion showing differences in plasma ceramides during the progression from non-diabetes to diabetes with and without microvascular disease. This aggregation of predictive information was accomplished by using the multiple regression analysis results, which incorporate the effects of individual covariates and allow multiple comparisons using the Bonferroni Method. Hence, the final models were obtained using the two composite predicted probabilities obtained using a panel of 11 individual ceramides versus the three ratios validated in other studies in the literature [25]. SPSS, Prism Plus 9, and Wizard 2 software were used for data and for graphical analysis.

**Table 1:** Clinical features, medication-use, and medication-adherence

Clinical features	Healthy	Diabetic		*P*-value*
	(*N*=51)	w/o AMI (*N*=150)	w/AMI (*N*=108)	
	Group 1	Group 2	Group 3	
**Gender; N (%)**				
Male	17 (33.3%)	52 (34.7%)	76 (70.4%)	<0.001
Female	34 (66.7%)	98 (65.3%)	32 (29.6%)	
**Age**				
Mean ± SD	35.8 ± 11.1	54.8 ± 12.3	65.1 ± 10.7	<0.001
**Ethnicity; N (%)**				
White	44 (86.3%)	108 (72%)	71 (66.4%)	0.027
Other	7 (13.7%)	42 (28%)	36 (33.6%)	
**Social Status; N (%)**				
A-B	47 (92.2%)	82 (54.7%)	34 (46.6%)	<0.001
C-E	4 (7.8%)	68 (45.3%)	39 (53.4%)	
**Physical Exam**				
**BMI, kg/m2**				
Mean ± SD	23.7 ± 3.3	29 ± 5.7	28.6 ± 5	<0.001
**Waist Circumference (cm)**				
Mean ± SD	81.8 ± 10.9	99.4 ± 14.2	103.4 ± 10.5	<0.001
**SBP (mmHg)**				
Mean ± SD	117.3 ± 12.9	135.6 ± 17.1	125.8 ± 17.6	<0.001
**DBP (mmHg)**				
Mean ± SD	77 ± 8.9	82.6 ± 9.8	77.6 ± 11	<0.001
**Heart Rate (bpm)**				
Mean ± SD	73.2 ± 10	82.2 ± 10.6	72.6 ± 9	<0.001
**Comorbidities**				
**Diabetes; N (%)**	0 (0%)	150 (100%)	108 (100%)	<0.001
**Hipertension; N (%)**	0 (0%)	99 (66%)	63 (84%)	<0.001
**Dyslipidemia; N (%)**	0 (0%)	58 (38.7%)	70 (93.3%)	<0.001
**Smoking; N (%)**				
Active	3 (5.9%)	12 (8%)	9 (10.6%)	0.014
Not Active	8 (15.7%)	56 (37.3%)	33 (38.8%)	
Never Smoked	40 (78.4%)	82 (54.7%)	43 (50.6%)	
**Drugs in use**				
**Oral hypoglycemic; N (%)**	0 (0%)	115 (76.7%)	64 (85.3%)	<0.001
**Insulin; N (%)**	0 (0%)	66 (44%)	19 (25.3%)	<0.001
**Antihypertensive; n/N (%)**	0 (0%)	99 (66%)	63 (84%)	<0.001
**Statins; N (%)**	0 (0%)	52 (34.7%)	70 (93.3%)	<0.001
**Ezetimibe; N (%)**	0 (0%)	1 (0.7%)	3 (4%)	0.098
**Fibrate; N (%)**	0 (0%)	10 (6.7%)	5 (6.7%)	0.137
**MORISKY (MMAS) Questionnaire**^a^				
Mean ± SD	-	1.7 ± 1.6	2.9 ± 1.7	<0.001

*BMI* Body Mass Index, *SBP* Systolic Blood Pressure, *DBP* Diastolic Blood Pressure, *SD* Standard Deviation

^a^Morisky test was used as a tool to assess patients adherence to drug treatment

MMAS <6: Poor Adherence/ MMAS 6-7: Medium Adherence/ MMAS>= 8: High Adherence

A = upper class, B = upper middle class, C = middle class, D = lower middle class, E = lower class

Fisher Exact Test; Kruskal-Wallis test

*P-Value signiifcant (<0.05)

## Results

### Baseline characteristics and clinical outcomes

 This study included healthy individuals and a broad spectrum of patients with diabetes of different ages, socioeconomic status, comorbidities, and medications in use (Table 1). Group 1 comprised younger, healthy individuals with a mean age of 35 years, high socioeconomic status, no comorbidities, and no medication usage. In contrast, Group 2 consisted of patients with a mean age of 54 years, multiple comorbidities including hypertension (66%) and dyslipidemia (38%), and all patients were diagnosed with diabetes and treated either with oral hypoglycemic medication or insulin. Group 3 comprised older patients with a mean age of 65 years, diagnosed with diabetes and an acute AMI, and who were on a higher regimen of cardiovascular disease medications. Group 1 exhibited an HbA1c mean value of 5.1%, as expected for healthy volunteers (Table 2).

**Table 2:** Diagnostic exams

Clinical Features	Healthy	Diabetic		*P*-value*
	(*N*=51)	w/o AMI (*N*=150)	w/AMI (*N*=108)	
	Group 1	Group 2	Group 3	
**Total cholesterol (mg/dL)**				
Mean ± SD	185.9 ± 33.4	192.8 ± 40	144.7 ± 34.2	<0.001
**HDL (mg/dL)**				
Mean ± SD	62.7 ± 18.2	49.7 ± 16.2	41 ± 11.1	<0.001
**LDL (mg/dL)**				
Mean ± SD	102.5 ± 32.6	110.6 ± 32.2	76.3 ± 28.6	<0.001
**Triglycerides (mg/dL)**				
Mean ± SD	113.5 ± 105.1	203.4 ± 178	181.9 ± 111.2	<0.001
**C-Protein Reactive (mg/dL)**				
Mean ± SD	1.5± 0.2	4.7± 0.5	4.2± 1.6	<0.001
**HbA1c (%)**				
Mean ± SD	5.1 ± 0.3	8.3 ± 2	7.6 ± 1.9	<0.001
**Hemoglobin (g/dL)**				
Mean ± SD	13.9 ± 1.3	14.1 ± 1.6	13.6 ± 1.6	0.044
**Insulin (mUI/mL)**				
Mean ± SD	10.7 ± 7.8	28.4 ± 25.7	21.9 ± 25.1	<0.001
**GFR (mL/min/1,73m2)**				
Mean ± SD	101.4 ± 22.4	99.9 ± 28.5	82.6 ± 24.4	<0.001
**Cardiac MRI**	**(*N*=51)**	**(*N*=114)**	**(*N*=45)**	*P*-value*
**Left Ventricle EF (%)**	#			
Mean ± SD	68.9 ± 4.2	65.4 ± 8	62.5 ± 10.4	0.138
**Right Ventricle EF **				
Mean ± SD	-	62.4 ± 6.6	63.7 ± 8.8	0.219
**LV End-Diastolic Diameter (cm)**				
Mean ± SD	4.6 ± 0.4	4.9 ± 0.6	5 ± 0.8	0.019
**LV Systolic Diameter (cm)**				
Mean ± SD	2.8 ± 0.3	3.1 ± 0.6	3.5 ± 1	0.001
**Retinography**	**(*N*=50)**	**(*N*=144)**	**(*N*=54)**	*P*-value*
Absent	50 (100%)	99 (69%)	35 (64%)	<0.001
Non-proliferative	0 (0%)	40 (28%)	15 (28%)	
Proliferative	0 (0%)	5 (3%)	4 (7%)	
6-Month Follow-up	**(*N*=50)**	**(*N*=144)**	**(*N*=54)**	*P*-value*
Quality of Life (EQ-5D)	5.6 ± 0.8	6.9 ± 1.5	7.0 ± 1.9	<0,001
MACCE	0 (0%)	1 (0.6%)	0 (0%)	0.126

*GFR* Glomerular filtration rate, *MRI* Magnetic resonance imaging, *EF* Ejection Fraction, *SD* Standard deviation

# Healthy group performed echocardiogram

*MACCE* Major cardiovascular and cerebrovascular adverse event

*P-Value signiifcant (<0.05)

Conversely, Groups 2 and 3 demonstrated HbA1c values of 8.3% and 7.6%, respectively—both consistent with diabetes. All laboratory results for Group 1 were within the normal range based on statistical analysis of a healthy population. In contrast, Group 3, while on treatment, exhibited a lower LDL-cholesterol level of 76.3 ± 28.6 compared to the other groups, and all patients in this group maintained normal renal function. Regarding cardiac cavity diameters, Group 3 had increased left ventricle diastolic and systolic heart dimensions.

Although the left ventricular ejection fraction was within the range considered normal (≥ 50%). Group 1 had no abnormalities observed in the retinography exam, while Groups 2 and 3 had about 30% of patients with non-proliferative microvascular lesions. Group 3 had twice as many patients with proliferative lesions as Group 2 (7% vs. 3.5%) (*p*-value < 0.001). In addition, in the six-month follow-up, only one MACCE was observed in Group 2, while no adverse events were reported in other groups.

### Analysis of ceramide kinetics

**Fig. 2:**
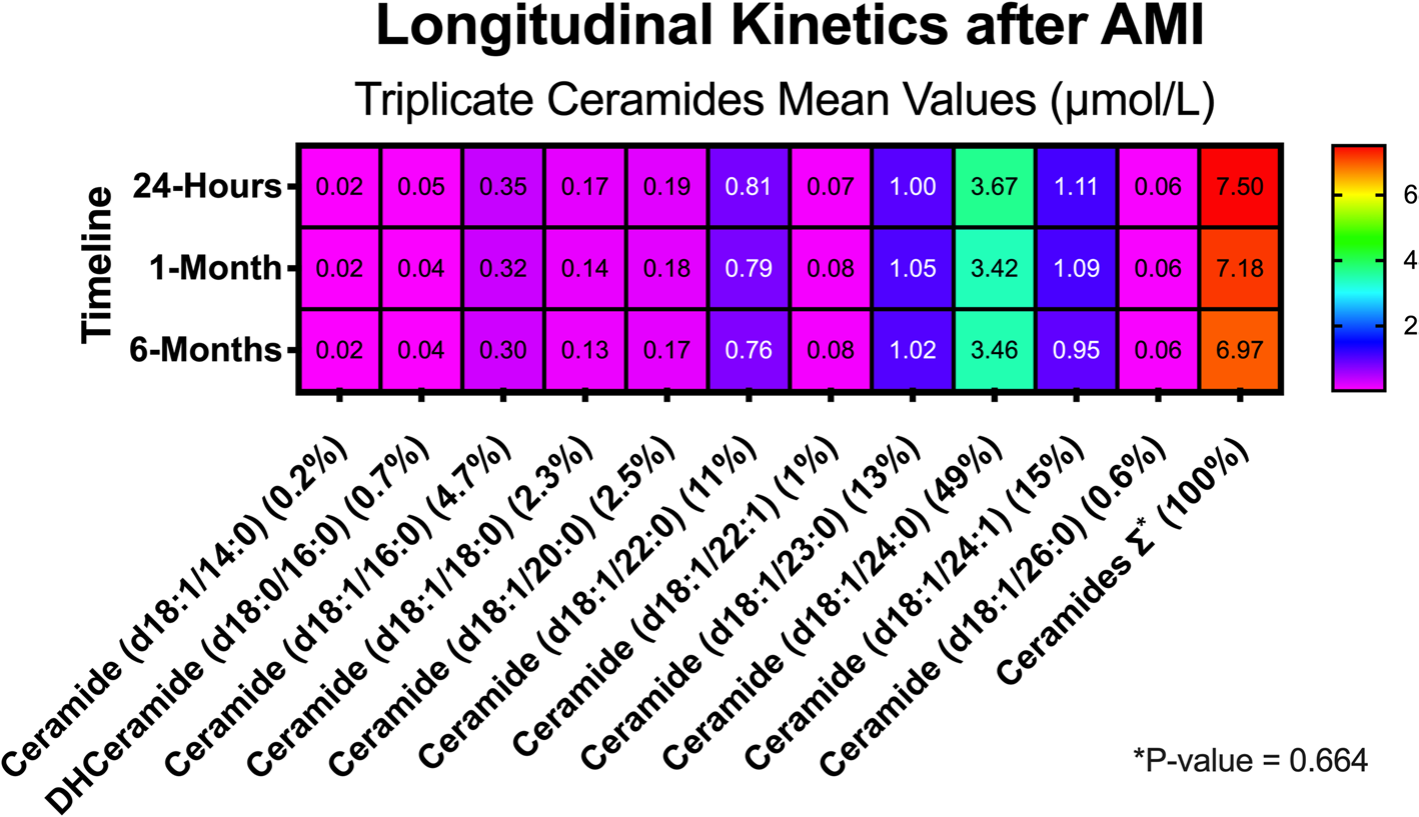
Heat map with longitudinal ceramide kinetic analysis (Group 3/*N* = 48 pts). *P -value = 0.664

The longitudinal kinetics of ceramides in Group 3 (*N* = 48 pts) demonstrated a non-significant increase in total plasma concentrations after AMI at 24-hour, one-month, and six-month follow-ups (*P* = 0.664) (Fig. 2). The analysis of all individual 11-ceramide panel kinetics indicated plasma stability, as detailed in Supplementary Table 1.

### Comparison of ceramide plasma concentrations among healthy and DM patients

**Fig. 3:**
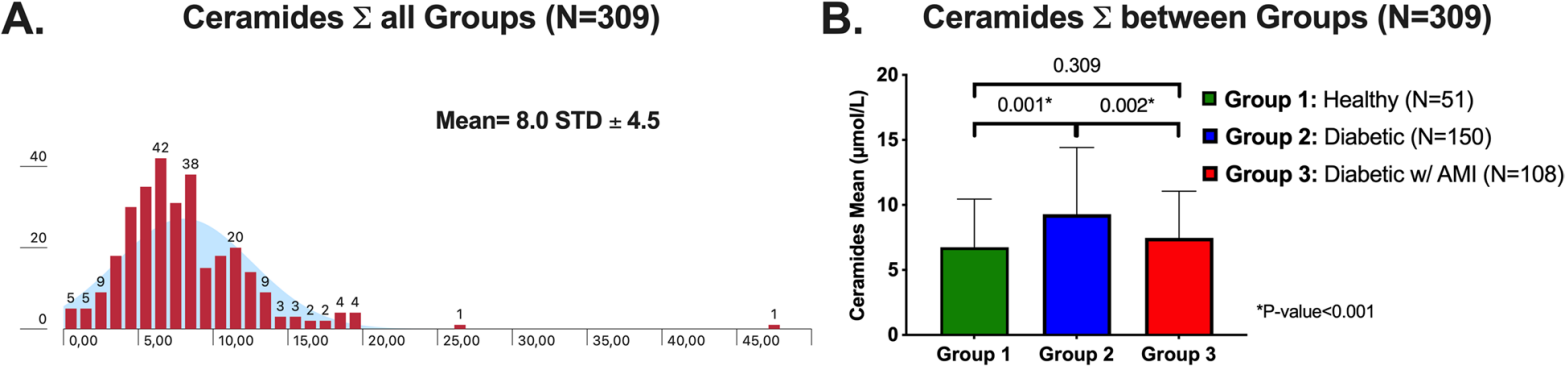
Differences between plasma ceramide concentrations

The total mean concentration of plasma ceramides across the entire cohort was 8.0 ± 4.5 µmol/L (Fig. 3A). The median plasma concentration of ceramides in diabetic patients (Groups 2 and 3, *N* = 258 pts) was 7.0 µmol/L, which differed significantly when compared to healthy individuals (non-diabetic group) (Group 1, *N* = 51 pts) at 6.3 µmol/L (*p* = 0.01) (Fig. 3B). These results confirm the association of these biomarkers with diabetes status.

### The spectrum of cardiac and retinal microvascular disease

**Fig. 4:**
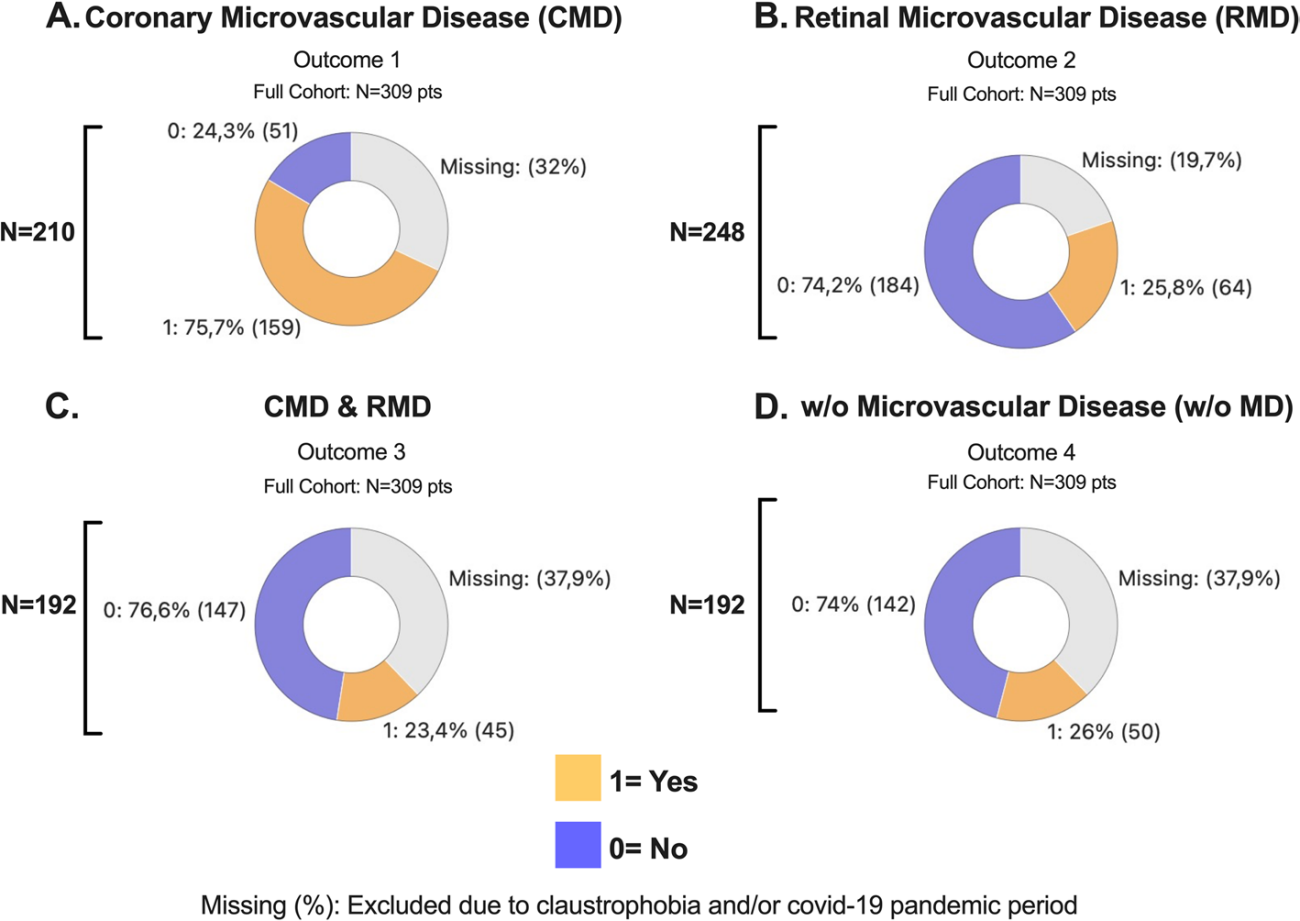
Entire cohort: main outcomes

A total of 159 CMR exams were conducted in diabetic patients (Groups 2 and 3), while 51 healthy individuals (Group 1) served as a control group, all showing normal echocardiographic and retinography results (total = 210 pts). CMD was diagnosed in 75.7% (159 pts) (Outcome 1), with a missing patient proportion of the entire cohort being 32%, as illustrated in Fig. 4A. In total, 248 retinography exams were performed, diagnosing RMD in 25.8% (64 pts) (Outcome 2), with a missing patient proportion of the entire cohort being 19.7%, as shown in Fig. 4B. CMD & RMD (Outcome 3) were observed in 23.4% (*N* = 45 pts) out of 192 patients, as depicted in Fig. 4C. Patients without microvascular disease (w/o MD) accounted for 26% (*N* = 50 pts) out of the 192 patients, as shown in Fig. 4D.

### Ceramides predict microvascular phenotypes

**Fig. 5:**
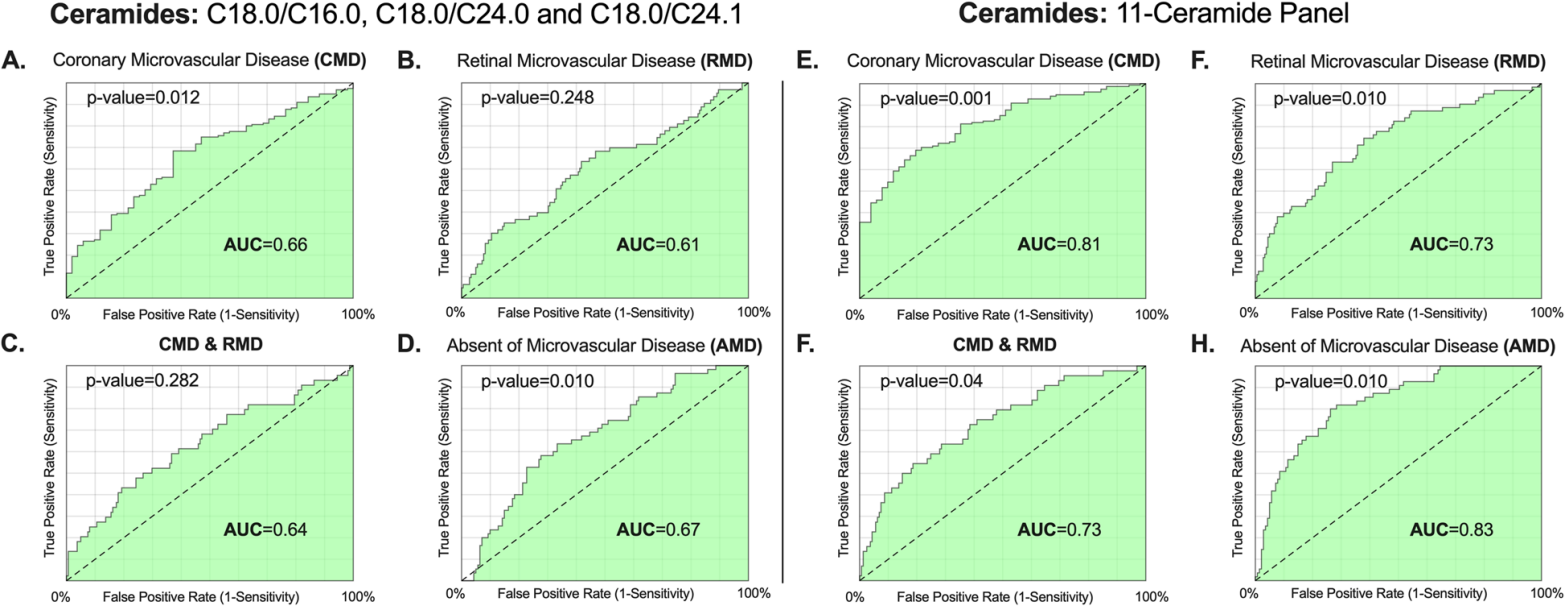
Roc curve analyses of three reference ceramide ratios (C 18.0/16.0; C 18.0/24.0 & C 18.0/24.1) *Versus* 11-ceramide panel

The three ratios identified in a large populational study, which are associated with diabetes according to the literature, were analyzed and compared with an 11-ceramide panel. The AUC using reference ceramide ratios for each outcome were: 0.66 (*p* = 0.012) for CMD, 0.61 (*p* = 0.248) for RMD, 0.64 (*P* = 0.282) for CMD & RMD, and 0.67 (*P* = 0.010) for w/o MD. However, the AUC values using the 11-ceramide panel showed significant improvement: 0.81 (*p* = 0.001) for CMD, 0.73 (*p* = 0.010) for RMD,0.73 (*P* = 0.04) for CMD & RMD, and 0.83 (*P* = 0.010) for w/o MD (Fig. 5).

### Differential ceramide profiles of retinal and cardiac microvascular phenotypes

**Fig. 6:**
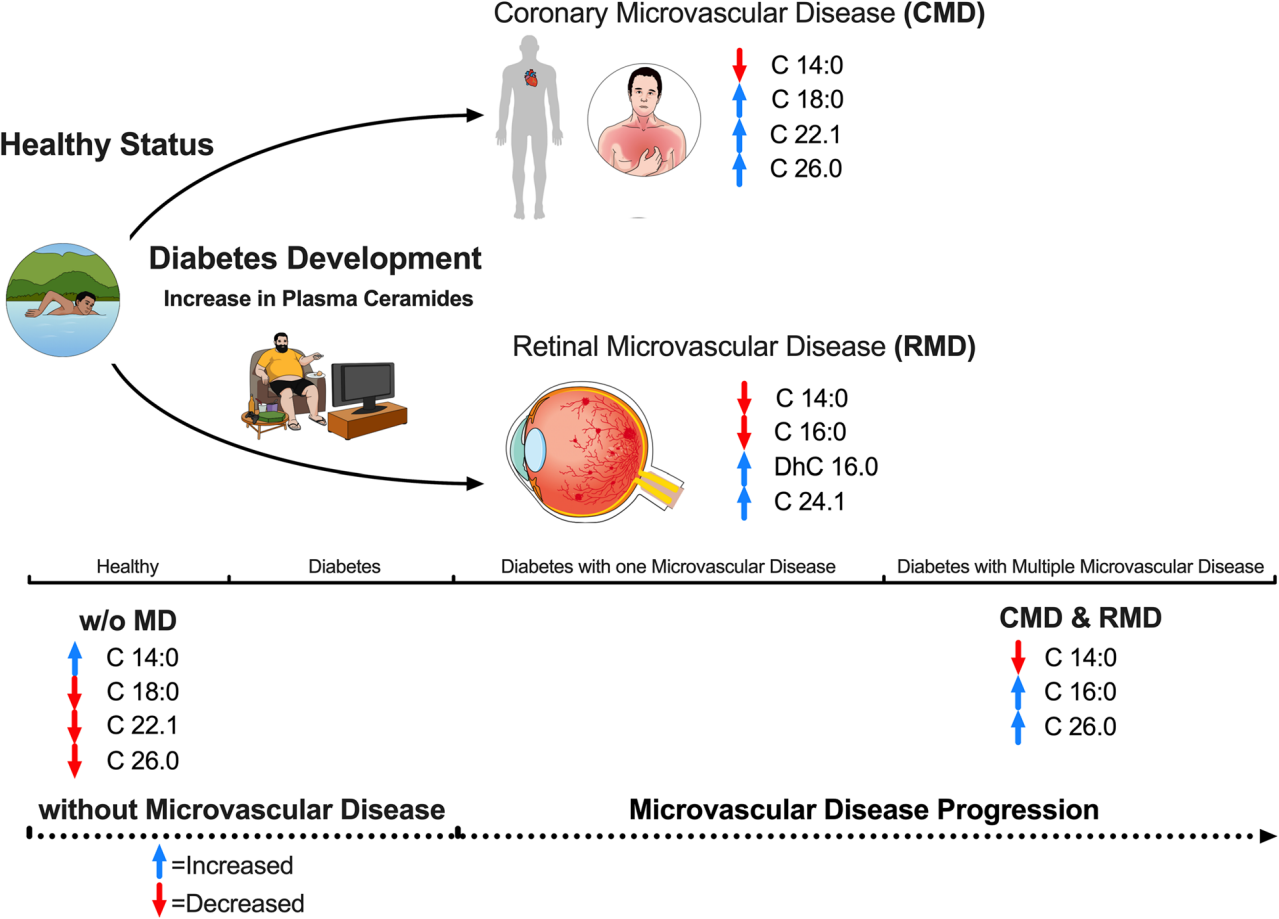
Visual conclusion of the study with the progression from non-diabetes to diabetes with microcirculatory disease and the signature patterns of biomarkers with significant statistical association

All four outcomes in our study were analyzed using the entire 11-ceramide panel. The different patterns of ceramides were demonstrated by their coefficients and p-values (Table 3) derived from the multiple logistic regression model. Patients with CMD had reduced C14.0 (p = < 0.001) and increased plasma levels of C18.0 (*P* = 0.009), C22.1 (*P* = 0.01), and C26.0 (*P* = 0.05). Patients with RMD exhibited reduced levels of C14.0 (*p* = 0.01) and C16.0 (*P* = 0.01) and increased plasma levels of DhC16.0 (*P* = 0.009) and C24.1 (*P* = 0.02). Patients with both CMD & RMD had lower levels of C14.0 (*P* = 0.03) and C16.0 (*P* = 0.05) and, in contrast, higher levels of C26.0 (*P* = 0.04). Finally, patients and healthy individuals w/o MD had higher levels of C14.0 (*p* < 0.001) and lower levels of C18 (*P* = 0.01), C22.1 (*P* = 0.007) and C26.0 (*P* = 0.02). Interestingly, C14.0 was only positively associated w/o MD. The study’s visual conclusion supports different trends of ceramides as a signature for each disease spectrum (Fig. 6).

**Table 3:**
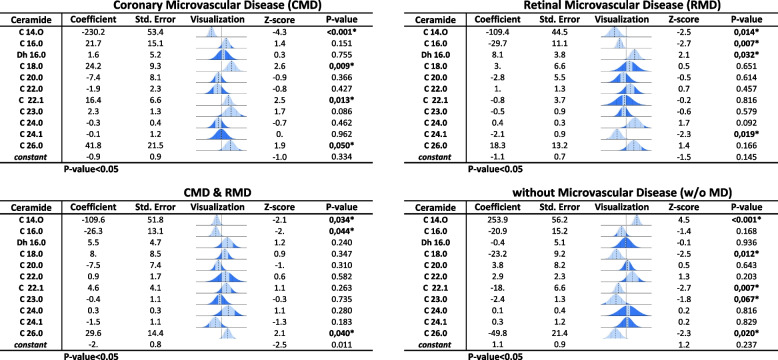
Logistic regression of 11-ceramide panel and all outcomes

## Discussion

Microcirculation is emerging as a major determinant of cardiovascular outcomes among patients, regardless of the presence or absence of DM [26]. Thus, this study aimed to bridge this gap and evaluate the effectiveness of using ceramide as a biomarker for microvascular disease.

The four outcomes analyzed not only cover the disease progression, but also identify distinct ceramide profiles that reflect core pathological disease stages, from healthy individuals to those in early or advanced stages of diabetic disease. The main findings in this study are as follows: Throughout the six-month observational period, the mean concentration of ceramides in plasma remained constant, higher levels of ceramides are found in diabetic patients, and ceramides identified in our previous study in macrovascular disease are also associated with the progression of microvascular disease.

Ceramides were previously identified by our group and others as tissue-based biomarkers linked to unstable atherosclerotic plaque [14, 27]. Ceramides are produced in response to hyperglycemia [28], tumor necrosis factor (TNF)-α signaling [29], NO-signaling, inflammation [30], and vascular FAT-redox state and are linked to poor cardiovascular outcomes [31]. As ceramide levels increase, they induce apoptosis and fibrosis [32]. Hence, ceramides are interrelated to cardiometabolic syndrome, diabetes, microvascular disease development, and mortality [33]. However, microvascular disease is often difficult to detect and therefore underdiagnosed [20].

The study was designed to identify variations in plasma ceramide levels among patients with and without various types of microvascular disease, which explains the intentional dissimilarity among our comparison groups. A key finding from this study is that distinct ceramide profiles are associated with each specific pathological territory of microvascular disease, whether occurring independently or in conjunction. Therefore, participants were categorized based on divergent health and disease conditions to document the natural progression of microvascular disease in diabetes. This categorization utilized noninvasive plasma biomarkers, providing valuable information for the risk stratification of these patients [34].

Common atherosclerotic risk factors often fail to accurately classify patients at risk of MACCE or microvascular disease [35]. Interestingly, LDL-cholesterol has been found to have limited predictive value for MACCE risk when included in models that use ceramides [36]. There is mounting evidence suggesting that circulating ceramides may predict the risk of ACVD, potentially even more effectively than LDL cholesterol [37]. Ceramides have emerged as a new category of biomarkers for cardiovascular disease. The Coronary Event Risk Test (CERT) is a validated method for predicting cardiovascular risk, relying solely on circulating levels of ceramide [38].

Previous studies have identified specific ceramide ratios associated with diabetes but not specifically with microvascular disease [24]. Within this study, it is noteworthy that the ceramide ratio C14.0 correlated with all outcomes. A CMD diagnosis was associated with increased levels of C18.0, C22.1, and C26.0. Conversely, there is a negative relationship between ceramide 14.0 and the large body of literature observing similar findings [39–41]. Additionally, an increase in ceramide levels has been shown to be detrimental to the retina, leading to inflammatory events, apoptosis, and retinal degeneration by disrupting the balance of cell death and survival [42].

Previous studies have demonstrated that a high ceramide ratio of C16.0 is strongly associated with MACCE [39, 43] and other metabolic defects that contribute to the pathophysiology of diabetes, such as insulin resistance. Furthermore, cholesterol dysmetabolism and diabetes can act synergistically to develop diabetic retinopathy [44]. C16.0 was found to be reduced in patients with RMD in this study and has been implicated in the development of obesity and type 2 diabetes [45].

Recent studies have identified poor cardiovascular health through lipidomic profiling. Moreover, a low level of ceramide C14.0 has been linked to poor blood pressure control, elevated total cholesterol, and increased fasting blood glucose [46, 47]. This study yielded similar findings, indicating that a high concentration of plasma ceramide C14.0 is a signature of a healthy status, while a low concentration signals a disease state. Epidemiological studies suggest that the risks of microvascular disease and ACS begin at the pre-diabetic stage [48]. Detection of pre-diabetes using plasma ceramides [24] might represent an opportunity to reduce the burden of both microvascular and macrovascular diseases through increased screening for vascular complications. This has critical implications in clinical practice, as prevention studies have demonstrated that those risks can be mitigated with lifestyle interventions [49]. Additionally, evidence is mounting that not only ceramides but also numerous other sphingolipid metabolites like sphingosine, sphingomyelin, and ganglioside act as pivotal regulators of insulin responses across various experimental contexts [50].

However, this study has several limitations that warrant mention. Firstly, some patients did not undergo heart and retina microvascular disease exams due to the risk of infection during the COVID-19 pandemic and/or claustrophobia during stress CMR [51]. The reported incidence of anxiety-related reactions during MRI ranges from 4 to 30% in the general population. Secondly, the present study observed a lower incidence rate of MACCE, and intermediate outcomes could not be associated with problematic outcomes, likely due to the high percentage of patients on lipid-lowering therapy. However, the study protocol was not predefined to evaluate MACCE, and the inclusion of this data could potentially have enhanced the value of our findings. Thirdly, the healthy group (Group 1) had heart function assessed only by echocardiogram. Lastly, the inclusion of patients with both type 1 and type 2 diabetes, although they represent a heterogeneous population with varied disease duration, metabolic control, and treatments, adds complexity to the study findings.

Altogether, the biological effect of microvascular disease on organ dysfunction is a crucial element in the progression of diabetic disease, and the development of noninvasive biomarkers capable of diagnosing microvascular disease at an early stage is critical to reducing disease progression. These findings support the idea that ceramide is a viable biomarker that can be used to refine the classification of diabetic microvascular disease.

## Conclusion

Plasma ceramides demonstrate distinct signatures associated with the presence of retinal and/or cardiac microcirculatory dysfunction in both the presence and absence of DM and coronary artery disease. Our findings may inform future studies investigating the links between circulating ceramides and microvascular disease.

## Supplementary Information

Supplementary Material 1.

## Data Availability

The datasets generated during and/or analyzed during the current study are not publicly available but are available from the corresponding author upon reasonable request.

## Abbreviations

DM: Diabetes mellitus
AMI: Acute myocardial infarction
CMR: Cardiac magnetic resonance
CMD: Coronary microvascular disease
RMD: Retinal microvascular disease
w/o MD: without Microvascular Disease
ROC: Receiver operating characteristic curve
C: Ceramide
MS: Mass spectrometry
MACCE: Major adverse cardiac and cerebrovascular events
T2D: Type 2 diabetes
ACS: Acute coronary syndrome
ADA: American diabetes association
MMAS: Morisky medication adherence scale questionnaire
SIMI: Stress-induced myocardial ischemia
SSFP: Steady-state free precession
PC: Phase contrast
MBF: Myocardial blood flow
CFR: Coronary flow reserve
HbaA1: Glycated hemoglobin
TNF: Tumor necrosis factor
